# Active Video Game Exercise Training Improves the Clinical Control of Asthma in Children: Randomized Controlled Trial

**DOI:** 10.1371/journal.pone.0135433

**Published:** 2015-08-24

**Authors:** Evelim L. F. D. Gomes, Celso R. F. Carvalho, Fabiana Sobral Peixoto-Souza, Etiene Farah Teixeira-Carvalho, Juliana Fernandes Barreto Mendonça, Roberto Stirbulov, Luciana Maria Malosá Sampaio, Dirceu Costa

**Affiliations:** 1 Postgraduate Program in Rehabilitation Sciences, Nove de Julho University, São Paulo, Brazil; 2 Physical Therapy Department, University of São Paulo, São Paulo, Brazil; 3 Santa Casa School of Medical Sciences, São Paulo, Brazil; Vanderbilt University, UNITED STATES

## Abstract

**Objective:**

The aim of the present study was to determine whether aerobic exercise involving an active video game system improved asthma control, airway inflammation and exercise capacity in children with moderate to severe asthma.

**Design:**

A randomized, controlled, single-blinded clinical trial was carried out. Thirty-six children with moderate to severe asthma were randomly allocated to either a video game group (VGG; N = 20) or a treadmill group (TG; n = 16). Both groups completed an eight-week supervised program with two weekly 40-minute sessions. Pre-training and post-training evaluations involved the Asthma Control Questionnaire, exhaled nitric oxide levels (FeNO), maximum exercise testing (Bruce protocol) and lung function.

**Results:**

No differences between the VGG and TG were found at the baseline. Improvements occurred in both groups with regard to asthma control and exercise capacity. Moreover, a significant reduction in FeNO was found in the VGG (p < 0.05). Although the mean energy expenditure at rest and during exercise training was similar for both groups, the maximum energy expenditure was higher in the VGG.

**Conclusion:**

The present findings strongly suggest that aerobic training promoted by an active video game had a positive impact on children with asthma in terms of clinical control, improvementin their exercise capacity and a reductionin pulmonary inflammation.

**Trial Registration:**

Clinicaltrials.gov NCT01438294

## Introduction

Asthma is a chronic inflammatory disorder characterized by airway obstruction associated with recurrent episodes of wheezing, shortness of breath, chest tightness and coughing[1].

The symptoms experienced during daily physical activities or the fear of triggering these symptoms often keep asthmatic children from engaging in physical exercise, which leads to a reduction in physical fitness [2,3,4]. Nevertheless, there is evidence that physical exercise is an important non-pharmacological component of the clinical control of asthma in both children[5] and adults [6]. Recent studies have demonstrated that aerobic exercise improves their exercise capacity [7] and reduces airway inflammation [8]. However, the treatment of a chronic disease that involves continual physical training can be discouraging. This seems especially important for children, since better performance and greater energy expenditure require an intrinsic motivation for physical activity [9].

Changes in lifestyle in the latter half of the 20^th^ century have raised concerns, since four fifths of children and adolescents do not follow public health guidelines regarding the recommended levels of physical activity [10,11]. Although new technologies have hugely affected the increasing sedentary lifestyle amongst young people, technologies, such as active video game systems with a high degree of energy expenditure [12], have also been employed therapeutically to stimulate activities amongst both pediatric and adult patients [13,14]. Studies involving obese adolescents have demonstrated the benefits of this therapeutic resource to improve body composition[14]. However, no previous studies have been carried out assessing the use of active video games for the rehabilitation of children with asthma.

The hypothesis put forth herein is that an active video game system can be as effective as treadmill training to improve clinical control and aerobic fitness in children with asthma. Thus, the aim of the present study was to determine whether aerobic exercise involving an active video game system improved asthma control, airway inflammation and exercise capacity in children with moderate to severe asthma.

## Materials and Methods

### Patients

This was a randomized, controlled, single-blinded clinical trial with 36 asthmatic children from a tertiary center specialized in childhood asthma, and the protocol was carried out in the University clinic (specialized in pulmonary diseases). All children presenting the following inclusion criteria were invited to participate: (i) diagnosis of asthma based on the guidelines of the Global Initiative for Asthma [1]; (ii) medical treatment for at least two months prior to the study; (iii) clinical stability (i.e., no exacerbation or change in medication in the previous 30 days); and (iv) not having participated in any regular exercise training program. The following were the exclusion criteria: (i) respiratory infection in the previous two months; (ii) inability to perform any test; (iii) diagnosis of heart disease; and (iv) an infection with fever (>37.5°C) in the previous two weeks.

The study was approved by the university ethics committee (Ethics Committee register- Nove de Julho University- 463982/2011) and registered with the clinicaltrials.gov (NCT01438294). All caregivers gave their written informed consent prior to inclusion of the children. All evaluations were carried out by an examiner who was blinded to the allocation.

### Randomization

Eligible children were randomly allocated to either a video game group (VGG) or treadmill group (TG). The Microsoft Excel program was used to generate a simple randomization sequence. Allocation was determined using numbered, sealed, opaque envelopes. Two envelopes were prepared for every participant. An envelope was chosen by the participant after the baseline measurements had been made. The whole randomization process was carried out by a researcher who was blinded and did not take part in the protocol.

### Experimental design

**Fig 1:**
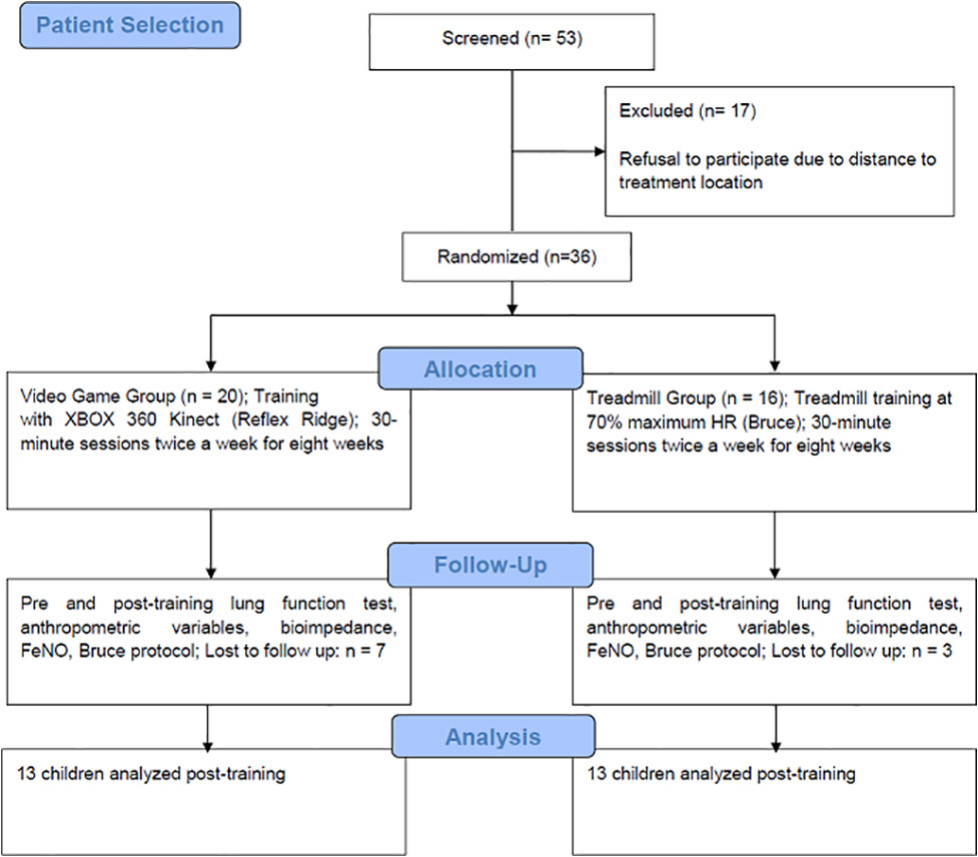
Flowchart of the study (in compliance with the CONSORT statement).

The participants were selected based on the eligibility criteria and randomly allocated to either the VGG (n = 20) or TG (n = 16). Before and after the training protocols, the participants answered questions on asthma control and were submitted to the FeNO, maximum exercise, tetrapolar bioimpedance and lung function tests. The children were submitted to all the assessments during the first week and began the training protocols the following week. The training period lasted eight weeks and involved two weekly 40-minute sessions (5 minutes of warming up, 30 minutes of training and 5 minutes of cooling down). Fig 1. Flowchart of the study.

### Video game training

The game “Reflex Ridge” from Kinect Adventure (XBOX 360 Kinect,) was used for training. A five minutes warm up period was carried out on a treadmill at 2 km/h prior to each session. The children then played the video game for 30 minutes (10 three-minute rounds with a 30-second rest interval between rounds) followed by a 5-minute cooling down on the treadmill again. The intensity was increased when the child successfully concluded a game level. A higher level required the child to carry out a greater number of jumps, squats, lateral movements and arm movements. Before and after each session, three measurements of the peak flow were made to detect exercise-induced bronchoconstriction (decrease ≥20% inpeakflow)[15].

### Treadmill training

A 5-minute warm up period was carried out on a treadmill at 2 km/h prior to each session, after which the exercise training was carried out for 30 minutes starting at 70% of the maximum effort determined during the maximum exercise testing. After training there was a 5-minute cooling down period. Before and after each session, three measurements of the peak flow were made. When the child maintained an exercise-intensity for two consecutive sessions with no increase in symptoms, the intensity was increased by 5% by increasing either the treadmill speed or inclination, as previously described[6].

For both groups, the heart rate, oxygen saturation and energy expenditure were monitored during training.

### Assessments

#### Exercise capacity

Maximum exercise testing was carried out on a treadmill using the Bruce protocol [16].The test was interrupted when the child reported maximal fatigue or when the heart rate reached 200 bpm[17]. During the test, the blood pressure and peripheral oxygen saturation were quantified and the child submitted to an electrocardiogram. The Borg scale was used to quantify the sensation of shortness of breath during effort and at rest[18]. The VO2 was calculated indirectly by ergometric program software (Cardiovex).

#### Pulmonary inflammation

The fraction of exhaled nitric oxide (FeNO) was used as a marker of pulmonary inflammation, as described elsewhere[19]. The participant was instructed to blow into the NIOX Mino device, following the recommendations of the American Thoracic Society/European Respiratory Society [20,21]. The participant was instructed to empty the lungs as much as possible and then breathe in as much as possible using the equipment, until maximum lung capacity, to minimize NO contamination from the ambient air. A nasal clip was used to avoid contamination from the sinus cavities. Expiration was carried out with a constant flow for at least six seconds.

#### Lung function

Lung function was determined before and after the inhalation of 400 μg of salbutamol. The technical procedures were carried out in a climate-controlled room as recommended by the ATS[22]. The normal values predicted were those proposed by Polgar and Promadhat (1971)[23]. A 12% and 200-mL increase in forced expiratory volume in one second (FEV_1_) in comparison with the baseline was characterized as a positive response to the bronchodilator.

#### Asthma Control Questionnaire (ACQ)

This questionnaire has seven items: five related to asthma symptoms, one on the use of short-acting ß2 agonists as a rescue drug, and one on the FEV_1_ before using the bronchodilator as a percent of the predicted value. The ACQ score is the mean of the item scores and ranges from 0 (completely controlled) to 6 (uncontrolled) obtained during a seven-day period. The cutoff point for controlled/uncontrolled asthma was 2 points. Thus the patients were classified as having their asthma controlled (< 0.75), partially controlled (0.75 to 1.5) or uncontrolled (> 1.5). The minimum clinically important difference was 0.5 on a seven-point scale [24,25,26].

#### Body composition

All participants were evaluated individually in the afternoon to avoid the influence of circadian changes. Height, weight and abdominal circumference were determined. Tetrapolar bioimpedance was measured using the Biodynamics model 310 with electrodes on the extremities of the right upper and lower limbs[27].

The body mass index (BMI) was determined as the weight divided by the height squared (Kg/m^2^). The waist circumference was determined with a tape measure at the navel level during expiration[28]. The *Anthro plus* program was used for the determination of Z scores using the standards established by the World Health Organization (WHO, 2007). BMI Z-scores were used to classify the children as obese or within the ideal weight range. Z scores between 2 and -2 were considered ideal[29].

#### Energy expenditure

Energy expenditure was measured using a biaxial accelerometer (SenseWear Pro activity monitor)[13] and calculated in metabolic equivalents (METs) as well as calories per minute. The SenseWear arm band was used during the exercise sessions as a comparative parameter of effort intensity. Energy expenditure at rest and during medium and maximum effort was determined during all sessions.

### Statistical analysis

The sample size of 24 children (12 in each group) was calculated based on the FeNO levels (primary outcome), considering a 90% power and an alpha of 5% to detect a difference of 14 ppb between the pre-training and post-training evaluations, with a standard deviation of 14.9 ppb based on previous findings^6^. Twelve children were included to compensate for possible sample loss. Ten children (seven in the VGG and three in the TG) did not return for the follow-up evaluations even after several requests, and thus the last recorded values were used (intention-to-treat analysis).

The Kolmogorov-Smirnov test was employed to determine the data distribution. Parametric variables were expressed as the mean ± standard deviation. Non-parametric variables were expressed as the median interquartile intervals (95% CI). A two-way ANOVA with Tukey’s post hoc test was used for the comparisons between the pre-training and post-training evaluations for the parametric data, and Friedman’s test with the post hoc Dunn test for non-parametric data. The unpaired t-test was used for the analysis of energy expenditure. The statistical analysis was carried out using the Minitab 14 statistical software package. The level of significance was set at 5% (p < 0.05). The size effect was calculated using Cohen’s d and the results were interpreted based on Cohen (2008)[30] as follows: small (0.21 to 0.49), medium (0.50 to 0.79) or large (≥0.80).

## Results

Ten children (seven from the VGG and three from the TG) withdrew from the study: four due to changes in the school schedule; three abandoned the study without explanation, two dropped out due to difficulties with the parents’ schedules and one moved to another city. Thus, 26 children completed the study (13 in each group). At the baseline, both groups were similar regarding asthma control, pulmonary inflammation, lung function, body composition and anthropometric data as well as exercise capacity. All patients were treated with budesonide and long acting β2-agonists and maintained the medication dosage throughout the study.

After training, a significant improvement in asthma control was found for both groups (p<0.05). However, only the children in the VGG showed a reduction in FeNO (p<0.05) (Table 1; p<0,05).

**Table 1:** Anthropometric data, body composition, lung function, pulmonary inflammation and asthma control in the sample before and after the exercise protocols. Data are presented as the mean±standard deviation except for ACQ6 (presented as the median) and 95% confidence interval (in parenthesis); FeNO = exhaled fraction of nitric oxide; ΔFeNO = (post-pre) FeNO; VGG = video game group; TG = treadmill training group; ppb = parts per billion; FEV_1_ = forced expiratory volume in first second; FVC = forced vital capacity; ACQ6 = Asthma Control Questionnaire (total score-lung function question).

	Pre VGG (n = 20)	Post VGG (n = 13)	p value	Pre TG (n = 16)	Post TG (n = 13)	p value
Gender F/M	7/13	4/9		7/9	5/8	
Age (years)	7.5±1.9			8.0±2.0		
Waist circumference (cm)	60.6±6.92	59.0 ± 6.88	0.45	62.80± 8.54	60.96± 6.53	0.23
BMI(Kg/m^2^)	15.40±3.40	15.63±2.31	0.83	16.44±3.04	16.39±2.74	1.0
Z-Score Weight	0.22(-0.46–1.57)	0.14(0–1.5)	0.69	0.5 (-0.13–1.12)	1(0.41–1.15)	0.85
Z-Score Height	0.94(0.1–1.61)	1.37(-0.07–2.1)	0.27	1.07 (-0.08–1.56)	1.59(0.57–2.4)	0.19
Z-Score BMI	-0.24 (-1.50–1.09)	-0.52(-1.21–0.45)	0.83	0.24(-1.64–1.02)	0.27(-1.3–1.02)	1.0
Lean mass (%)	88.2±7.3	88.7±5.4	0.82	84.4±8.7	85.8±8.5	0.20
Fat mass (%)	11.7±7.3	11.2±5.4	0.82	15.6±8.7	14.1±8.5	0.20
FEV_1_%	73.0±13.0	86.5±18.1	0.06	76.4±16.2	79.7±13.4	0.42
FEV_1_/FVC %	83.5±16.8	90.2±10.8	0.07	84.5±9.5	85.0±9.4	0.16
FeNO (ppb)	35.5±19.7	23.3±10.9*	0.04	31.7±15.7	29.3±21.5	0.64
ΔFeNO (ppb)	-13.2 (8.22)			-2.3 (7.44)		
ACQ6	1.71 (0.83–2.66)	0.25 (0–0.58) *	0.001	1.16 (0.58–1.66)	0.25 (0.08–0.5) *	0.01

*p<0.05 = compared with intragroup at baseline.

The total mean energy expenditure was measured during the training protocols using a biaxial accelerometer and the data presented in metabolic equivalents (METs) and calories per minute (cal/min). No difference was found between the groups regarding the mean energy expenditure, but a significantly higher maximum metabolic expenditure was found in the VGG (p<0.05; Table 2). Interestingly, the total energy expenditure was also higher in the VGG (Table 2; p<0.05).

**Table 2:** Energy expenditure during training for both groups in the training sessions. The data are presented as the average±standard deviation of all sessions. VGG: Video Game group, TG: treadmill group; cal/min = calories per minute; MET = metabolic equivalent of tasks.

	VGG	TG	p value
Energy expenditure-rest (cal/min)	1.44± 0.48	1.10± 0.07	0.06
(METS)	1.92± 0.63	1.26± 0.07	0.05
Energy expenditure-minimum (cal/min)	2.64 ±0.65	2.76± 0.66	0.58
(METS)	3.39 ±0.69	3.65 ± 0.87	0.30
Energy expenditure-maximum (cal/min)	7.31 ±1.64*	5.68 ±1.27	<0.001
(METS)	9.37 ± 1.99*	7.51 ±1.69	<0.001
Energy expenditure-mean (cal/min)	4.43 ±1.24	4.14 ± 0.81	0.31
(METS)	5.66±0.89	5.48± 1.08	0.49
Total energy expenditure (cal)	159.9 ± 41.6*	133.3± 32.1	<0.01

*p<0.05 = compared with TG

**Fig 2:**
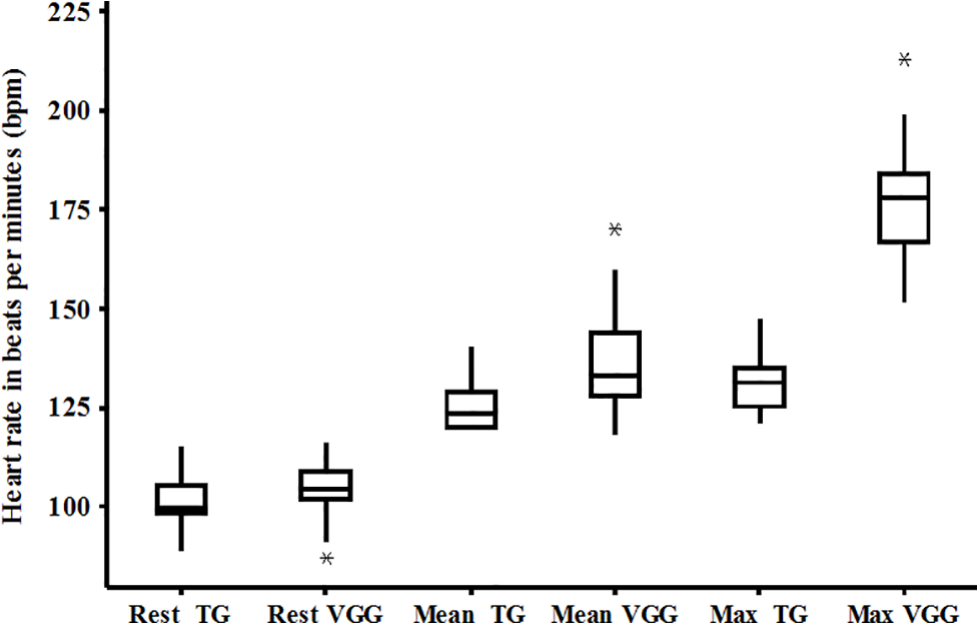
Heart rate response during training for both groups; the data presented in the box plot and boxes represent the 25th-75th percentiles; the lines inside the boxes represent the medians and the bars represent the 10^th^ and 90^th^ percentiles.

Fig 2 displays the heart rate (HR) at rest and the cardiac response during the training sessions and both were similar for the two groups. However, the percentage of the predicted maximum HR achieved during the training session was significantly higher in the VGG when compared to the TG (90.5% vs. 65.2%, respectively; p<0.01).

Both groups exhibited an improvement in maximum aerobic capacity after training (p<0.05; Table 3); however, the size effect was higher in the TG (1.6) when compared to the VGG (0.7). Similar results were observed for other variables during maximum exercise testing, such as exercise duration, velocity and distance. Regarding the cardiovascular variables, the VGG reached a greater percentage of the predicted maximum HR than the TG in the post-training evaluation, and both groups exhibited a significant increase in maximum double product.

**Table 3:** Data from the maximal exercise testing. The data are presented as the mean±standard deviation; VGG = video game group; TG = treadmill training group; VO_2_ = maximal aerobic capacity; HR = heart rate; double product = HR*blood pressure; The size effect was calculated using the Cohen method and classified as small = 0.21–0.49; medium = 0.50–0.79; and large = >0.80 as previously described (Cohen 1988).

Physical capacity	Pre VGG (n = 13)	Post VGG (n = 13)	Size effect	Pre TG (n = 13)	Post TG (n = 13)	Size effect
Time (min)	9.4±1.4	10.9±1.4*	1.07	9.7±1.4	12.5±1.0*	2.1
VO_2_ (mlO_2_/kg/min)	36.6±5.9	41.2±4.8 *	0.7	37.7±4.7	45.7±5.0*	1.6
Velocity (km/h)	5.9±0.8	6.5±0.6*	0.8	5.9±0.8	7±0.5*	1.3
Distance (m)	561.6±126.9	703.3±148.3*	0.9	607.6±148.5	895.8±143.4* ^#^	1.9
Rest HR (bpm)	102.0±3.86	100±2.8*	0.3	100.3±5.6	99.1±3.8	0.13
Max HR (bpm)	191.7±26.3	211.7±19.2	0.8	191.30±18.27	190.53±10.66^#^	0.0
% of max HR	96.0±12.7	103.2±8.6*	1.0	96.41±10.70	96±7.80^#^	0.1
Max double product	19887±5312	22947±3885*	0.7	22139±3049	25759±4154*	0.4

*intra-group p<0.05 before and after protocol

#inter-group p < 0.05 ANOVA pos hoc Tukey.

## Discussion

The present study clearly demonstrated that the exercise training using an active video game system was efficient in improving clinical control and aerobic fitness and in reducing lung inflammation in children with moderate to severe asthma. Moreover, the energy expenditure during video game training was higher than that achieved with aerobic training on a treadmill. Thus, active video games seem to be an interesting exercise for asthmatic children and are probably more attractive to the pediatric population.

The main goal of asthma treatment is to achieve and maintain good clinical control^1^. The present findings support the use of aerobic training combined with medication to achieve such goals and are in agreement with data reported in previous studies [7,31] regarding the improvement in clinical control through enhancing aerobic capacity. Moreover, the present study contributes further to the literature by demonstrating that an active video game can be used to improve physical fitness and clinical control in asthmatic children. Dogra et al[32] (2011) evaluated improvements in the clinical control of asthmatic patients using the ACQ-6, which was also employed in the present study. At the baseline, some patients were considered partially controlled or uncontrolled (ACQ>0.75). However, all patients from both groups were classified as having controlled their asthma after the exercise protocols (ACQ<0.75).

To the best of our knowledge, only three studies have evaluated the anti-inflammatory effects of aerobic training in asthmatic patients [6,33,34]. Moreira et al. (2008)[33] and Bonsignore (2008)[34] evaluated the effect of aerobic training in asthmatic children and found no changes in FeNO. In contrast, Mendes et al. (2011)[6] found a significant reduction in FeNO amongst asthmatic adults after exercise training. The divergence amongst these studies may be explained by differences in the exercise training and in the severity of the disease. Moreira et al. (2008)[33] and Bonsignore et al. (2008)[34] did not report any improvement in the exercise capacity of the patients, while this was observed in the present study and in Mendes’ study. Moreover, both studies also found a reduction in the FeNO levels, suggesting the importance of an increase in aerobic fitness to reduce airway inflammation [35,36].

It has been reported that approximately 50% of preschool children do not carry out even the minimum level of physical activity recommended by the American Academy of Pediatrics (≥60 minutes/day)[36]. This incidence may be even higher amongst children with asthma due to exercise-induced bronchospasm, which is reported to affect up to 90%[37]. Fun is the main driving force behind the practice of physical activity for children, and most of them spend an average of 65 minutes/day playing video games[38]. Thus, active video games would probably lead to a substantial reduction in this sedentary behavior and consequently increase the adherence to physical training amongst children with asthma.

To the best of our knowledge, aerobic exercise is the only type of physical activity that benefits asthmatic patients[39] and such activities should involve large muscle groups and be carried out for at least 20 minutes [40]. The treadmill has been the equipment most frequently employed for aerobic exercise amongst asthmatic adults and children [5,6]; however, the treatment of patients with chronic diseases with physical training can be discouraging [9]. The active video game Kinect/Reflex Ridge seems to be an interesting option because is an interactive game and requires full body movement, with squats, jumps and lateral movements. In the present study, activity was carried out for 30 minutes and achieved energy expenditure as high as 9.3 METs. Sustained activities reaching levels higher than 6 METs are considered vigorous and only the console type of video game has been previously evaluated in terms of energy expenditure, ranging from 3 to 6 METs in the household environment[41], where the proper stimulus is not always employed. According to Baquet et al. (2010) [42], exercise intensity should surpass 80% of the maximum HR to improve the aerobic performance of children without respiratory disorders. In the present population, the mean exercise intensity was 65.2% and 90.5% of the maximum HR in the treadmill and video game groups, respectively.

Kuys et al. (2011) [13] used a video game system as an exercise training tool in a hospital setting to improve aerobic fitness in adults with cystic fibrosis. However, no previous study has evaluated the use of active video games for aerobic training in children with asthma. The main proposal of the present study was to evaluate the effect of such exercise in a hospital setting, since most asthmatic children experience exercise-induced bronchoconstriction during exercise. However, the active video game seems to be a safe strategy, since no patients demonstrated a reduction in peak flow ≥20%.[15]

The motivation and intensity maintenance with video game training led to an improvement in the aerobic capacity of the patients. These findings are in agreement with data reported by Donovan et al. (2012) [41] and suggest that active video games could be used to improve aerobic capacity in sedentary individuals. However, Baranowski et al. (2012) [43] and Le Blanc et al. (2013)[44] reported that an active video game system did not increase the level of physical activity in children. Therefore, it seems that the type of game chosen for aerobic training and motivation are important issues to be considered.

Smallwood et al. (2012)[45] found that the energy expenditure using the XBOX Kinect was higher than with other video game systems. The authors attributed this finding to the fact that XBOX does not have any type of control device, which allows the player to carry out activities with larger movements. According to Mellecker et al (2013)[46], motivation is an important factor to increase energy expenditure during video game playing. The higher energy expenditure (>6 METs) observed in the present study was probably achieved due to the clinical setting and the constant motivation on the part of the physiotherapist during the sessions[47].

Both groups showed improvements in aerobic capacity during maximum exercise testing after the training protocols; however the TG presented better performance. Exercise on the treadmill was continuous, whereas exercise using the video game was performed at higher intensity (demonstrated by the HR; Fig 2) followed by short rest periods, similar to interval training. Two previous studies reported that continuous training led to a linear increase in peak VO_2_ while interval training also led to such an increase but not with the same linearity [42,48]. Another factor that could have contributed to the result of the TG was the specificity effect in exercise physiology.

The main limitation of the present study was due to the use of the SenseWear Pro Active device, which can underestimate the energy expenditure during vigorous physical activities [49]. This accelerometer seemed quite precise for the evaluation of energy expenditure during the supervised activities in the present study; however, the VGG achieved higher maximum expenditure.

Another limitation is the intensity of the effort in the video game, which cannot be individualized[50].

## Conclusion

The present findings strongly suggest that the aerobic training promoted by an active video game has a positive impact on children with asthma in terms of clinical control, improvement in their exercise capacity and a reduction in pulmonary inflammation.

## Supporting Information

S1 CONSORT Checklist CONSORT Checklist. (DOC)

S1 Protocol Trial Protocol. (PDF)
