# Supplementary material for: Active Video Game Exercise Training Improves the Clinical Control of Asthma in Children: Randomized Controlled Trial
Source: PLoS One. 2015 Aug 24;10(8):e0135433. doi: 10.1371/journal.pone.0135433 (PMC4547724; doi:10.1371/journal.pone.0135433)
Supplement: S1 Protocol — (PDF) [file pone.0135433.s002.pdf]

Physical, functional and quality of life of children with bronchial asthma submitted to a pulmonary rehabilitation program

Summary

What is known is that physical activity in children with asthma improves exercise capacity, improves quality of life, but there is no evidence of a beneficial effect on lung function and thoracoabdominal configuration thereof for technical and / or technological difficulties in assess these variables.

Based on studies that applied pulmonary rehabilitation programs in asthmatic children, we opted for the age group 5-11 years old, 16 weeks protocol duration at a frequency of twice a week and duration of the 50-minute session.

When it comes to the pediatric population must never forget the maxim that the child is not a small adult and therefore a rehabilitation program for this population should be adequate physiological and psychological needs of the same so that the grip is satisfactory and the desired results can be achieved.

The proposed program consists of fun activities that address the recommendations of the American Association of Cardiovascular and Pulmonary Rehabilitation [1] and the British Society of Thorax[2] with strength training and endurance of upper and lower limb and respiratory muscle training associated with an aerobic workout with emphasis on members below.

Introduction

Asthma is a chronic respiratory disease caused by an inflammation of the airways that manifests clinically with recurrent episodes of cough, dyspnea, wheezing and chest drawing. These episodes are related to an obstruction to airflow that is partly reversible.
Despite the difficulty to diagnose asthma in children, there is evidence to suggest that half of all cases of childhood asthma are diagnosed until 3 years of age and 80% of all cases of asthma up to 6 years, which is in 1/3 the first symptoms begin before the child is one year old. In children there is a predominance of males, ranging from 3: 2 to 2: 1. This supremacy is related to possible increased production of IgE and the higher tone of the airways, which are also closer in boys. The index shall be 1: 1 between 10 and 12, when the ratio diameter / length becomes the same for both sexes, when changes in the size of the chest in children, which does not happen with the girls. In adulthood begins to occur predominance of female [3] sex.
Overall the frequency of asthma declared doubled in the last twenty years, in part by increasing the number of real cases, as the best recognition of the disease by the medical community. The difficulty in comparing epidemiological data from one country to another, and sometimes from one region to another, motivated the development of a major international research, study ISAAC - "International Study of Asthma and Allergies in Childhood". In its first phase, through a simple and validated questionnaire, with few questions and self-administered, this study evaluated 304,796 children from 42 countries, between 6-7 years old and 463,801 teenagers in 155 centers in 56 countries between 13-14 year old. This research allowed us to distinguish three groups of countries according to prevalence rates of asthma: low (less than 5%), medium (5-6%) and strong (over 10%). In this study, Brazil was ranked 8th, with a prevalence of 20%.

Several factors may be contributing to the increase in childhood asthma. Among them, the increased survival of preterm fetuses with lungs not fully developed; increasing the number of smokers pregnant women, which increases the likelihood of low birth weight and reduced lung capacity in the newborn. Furthermore, it is a well known fact that exposure to cigarette smoke in uterus is capable of altering the growth of the airways and lungs of the fetus, which increases both the resistance and the risk of wheezing in the early stages of life .
All children with asthma should submit to the pulmonary function tests, as soon as possible. Above four years of age are able to perform maneuvers

with reasonable success. Pulmonary function tests help in the diagnosis and monitoring of disease .
The most common measures and easier to obtain are those forced expiratory maneuver through: forced vital capacity (FVC), forced expiratory volume in one second (FEV1), the FEV1 / FVC ratio and the maximum-average forced expiratory flow in vital capacity (FEF 25-75%).
The reduction in size and consequent increase in airway resistance determining all maximal decrease of expiratory flow, including peak expiratory flow (PEF) than in acute asthma in children may be smaller than 40 l / min. Still occur decrease in volumes expired a function of time, premature airway occlusion, pulmonary hyperinflation, increased work of breathing with changes in muscle performance, in the chest and biomechanical changes in the ventilation-perfusion ratio with change in the partial pressure of blood gases.
The biomechanical changes from asthma justify therapeutic interventions. Systematic reviews carried out between 2006 and 2007 7.8 evaluating the use of respiratory muscle training (RMT) and breathing exercises, alone or combined, have shown little evidence of benefit in asthma patients. Nobrega et al 2008 used TMR and breathing exercises in asthmatic children and shown to be effective as adjuvant therapy in the treatment of asthma with an increase in maximal respiratory pressures and Peak flow values.

Inflammatory marker as exhaled nitric oxide (FeNO) has been used for the diagnosis of asthma, and the cutoff value in these cases is 20 ppb, patients with respiratory symptoms associated with a FeNO > 20ppb may be diagnosed as asthmatic. Another advantage of this technique is that the Association of subclinical Asthma with elevated exhaled nitric oxide levels may indicate the need for a steroids treatment.
Whereas the exercise is strongly associated with anti-inflammatory effects, FeNO may be a sensitive parameter to detect its anti-inflammatory effect in the airways of children with asthma.
In terms of physical capacity and response to exercise, in theory, the vast majority of children with asthma should present a normal or near the physical capacity of children of the same age, but the restriction imposed by parents and some professionals makes them have a sedentary lifestyle and reduction of fitness. The poor physical performance of these children may also be related to poor condition and nutritional myopathy by use of oral corticosteroids.
According to the American Thoracic Society (ATS), pulmonary rehabilitation (PR) is defined as a multidisciplinary program of care for patients with chronic respiratory failure in order to optimize the autonomy, physical and social performance of these patients. The difficulty of treating

a pediatric population is to know in depth the characteristics of this population, tailor the language to be employed, finding methods for accession, gain their trust and emphasize the educational part to stimulate self-care and self-estima.

Repeated physical activities with varying intensity, which last a few seconds, interspersed with short periods of rest, are more appropriate for children because beyond them spontaneously prefer activities with high recreational component and a lot of variety, we explore more the anabolic effects of physical exercise. This fact justifies the investigation of the effects of recreational physical activity in asthmatic children.

As technology has evolved in favor of movement and physical activity and how studies with active video games comes this decade, proving to be attractive and with great influence in energy expenditure, draws attention to the possibility that application also in RP for asthmatic children. It is noteworthy that by the time this tool was used only in healthy individuals and therefore there is no evidence that this instrument can help the fitness of children or patients with respiratory diseases.

Based on this, we propose a program of recreational physical activities, with interactive video game for children that fall within the recommendations of the American Association of Cardiovascular Rehabilitation and Pulmonar especially targeting the aerobic training with emphasis on the lower limbs, as recommended by the British Society the Thorax.

OBJECTIVE

Evaluate the effects of a playful physical training program in pulmonary inflammatory process, quality of life and physical and respiratory functional evaluation variables of asthmatic children 5-11 years.

MATERIALS AND METHODS

Type of study: a randomized, control, blind clinical trial (children with asthma compared with healthy children)
The study will be conducted in the respiratory functional evaluation laboratory at the University Nove de Julho- UNINOVE, located at Av. Francisco Matarazzo, São Paulo-SP. Patients with asthma will be forwarded by two pediatric pulmonologists and the control group will be recruited by various means such as schools, family, friends and others.
Will be submitted to the COEP UNINOVE and will be recorded in clinicaltrial.gov

Inclusion criteria:

Ages 5 to 11 years
Having a diagnosis of asthma according to the criteria of the National heart, lung, and blood institute.
Not be included in any regular physical activity program or pulmonary rehabilitation.

Exclusion criteria:

Receiving theophylline or aminophylline and oral corticosteroids in the last 30 days
Have developed respiratory infection in the last two months
Have done inhalation bronchodilator in less than 12 hours before the
Inability to perform any of the tests
Have heart disease of inflammatory origin, congenital or ischemic
Being in the presence of an infectious process with fever
Not agree with the informed consent and informed and / or assent

Control group Inclusion criteria:

Ages 5 to 11 years
Not perform regular physical activity

Control group of exclusion criteria:

Have chronic lung disease such as cystic fibrosis, asthma, bronchial dysplasia.

Have heart disease of inflammatory origin, congenital or ischemic
Being in the presence of an infectious process with fever
Not agree with the informed consent and informed and / or assent

Experimental Protocol

There will be two evaluations, one before starting the program and after the end of it. These evaluations will consist of several tests, they are:

1) Exhaled Nitric Oxide (FeNO)
2) Shuttle walk test (ISWT)
3) Spirometry
4) Three minutes step test (ST3)
5) Heart rate variability (HRV)
6) Exercise test (Bruce protocol)
7) Pediatric Asthma Quality of life Questionnaire (PAQLQ)
8) Asthma control questionnaire (ACQ6)
9) Anthropometric data- bioimpedance

Exhaled Nitric Oxide FeNO

The fractional concentration of expired nitric oxide (FeNO) will be measured in the sitting position with the NIOX Mino™ equipment (Aerocrine, Sweden), following the guidelines of the American Thoracic Society (ATS). The test will be performed with the subject emptying the lungs until expiratory reserve volume, followed by inspiration through the oral cavity (with steps taken to eliminate the possibility of contamination) until achieving total lung capacity. The subject then exhaled slowly through the mouth piece maintaining a steady flow. After 1 minute 40 seconds,
a reading was made of FeNO in parts per billion. The procedure was performed with a nasal clip to avoid contamination from air in the sinus cavities.

Shuttle Walk Test

The shuttle walk test (ISWT) has progressive loading and 15 levels. The subject will be instructed to walk quickly at increasing speeds over a course of 10 meters delimited at each end by a cone, which the subject walked around. An audible beep signaled the change in level and increase in speed during the test. The test will be stopped when the subject was unable to reach the end of the 10-meter course by the time of the beep. Dyspnea upon exertion and at rest will be measured using the Borg Scale. Respiratory rate, heart rate, and saturation of peripheral oxygen will be also measured. Two tests will be conducted, with a 30-minute interval between the tests. The trial on which the greater distance traveled will be used for analysis.

Spirometry

Lung function will be determined before and after the inhalation of 400 µg of salbutamol (Easy One™, USA). The technical procedures will be performed in a climate-controlled room, as recommended by the ATS. Predicted normal values were those proposed by Polgar and Promadhat (1971). A 12% and 200-mL increase in forced expiratory volume in one second (FEV_1) in comparison to baseline will be characterized as a positive response to the bronchodilator.

Three Minutes Step Test

The subject will be instructed to climb up and down a single rung (15 cm in height) within a period of 3 minutes. Dyspnea, heart rate, and oxygen saturation (SpO2) will be recorded. Testing will be performed twice with a 30-minute interval, and the trial with the greater number of steps will be used for the analysis.

Heart rate Variability

The activity of the sympathetic and parasympathetic nervous systems will be collected by means of HRV analysis in the supine, seat position and during Bruce test, the most stable sections containing 256 points within 3 to 10 min will be selected using a Polar® S810i monitor. Will be analyzed time domain, frequency domain and non linear variable.

Exercise Testing (Bruce Protocol)

The stress test provides information on exercise capacity and facilitates access to pathophysiological characteristics, effectiveness of medications and the potential risk of disease. The child has physiologically underdeveloped knee extensors and for this reason the treadmill test is preferred to cycle ergometer in young children (pre -school 4-6 years). The Bruce protocol was developed for adults, but has been widely applied to children from 4 years old.

Pediatric Asthma Quality of life Questionnaire (PAQLQ)

The questionnaire of quality of life related to health is the most widely used instrument for childhood asthma, the PAQLQ is the best known and validated questionnaire for the Brazilian population and can be used in children 7-17 years. The PAQLQ consists of 23 questions divided into three domains: physical activity limitations (five questions), symptoms (10 questions) and emotions (eight issues). The responses are measured using a 7-point scale, where 1 indicates the maximum loss and 7 no harm.

Asthma Control Questionnaire (ACQ6)

This questionnaire has seven items: five related to asthma symptoms, one on the use of short-acting ß2 agonists as a rescue drug and one on FEV_1 before bronchodilator in percent of predicted. The ACQ score is the mean of the item scores and ranges from 0 (completely controlled) to 6 (uncontrolled) obtained in a seven-day period. The cutoff point for controlled/uncontrolled asthma was 2 points. Thus, the patients were classified as having their asthma controlled (< 0.75), partially controlled (0.75 to 1.5) or uncontrolled (> 1.5). The minimum clinically important difference was 0.5 on a seven-point scale.

Anthropometric data- bioimpedance

Height, weight and abdominal circumference will be determined. Tetrapolar bioimpedance will measured using the Biodynamics™ model 310 (Biodynamics Corporation Seattle WA, USA) with electrodes on the extremities of the right upper and lower limbs.

Experimental procedure

There will be two evaluations, one before starting the program and after the end of it. These evaluations will consist of tests above like anthropometric variables, heart rate variability and assessment of quality of life and physical capacity:
Children will be drawn to participate in one of two types of training and should achieve during the proposed activities heart rate equivalent to 70% of maximum heart rate achieved in the stress test.

Group 1: Aerobic training in the Treadmill (control group) - the gold standard
The training will be done on the treadmill with heart rate monitor with intensity required to meet 70% of maximum heart rate achieved in maximal test for thirty minutes.
• Evaluation of energy expenditure by the SenseWear Pro activity monitor (METS)

Group 2: Aerobic training with video game (XBOX 360 + Kinect)
• Training with video game will be done with heart rate monitor with intensity required to meet 70% of maximum heart rate achieved in maximal test for thirty minutes. Will use the Kinect adventure game “Ridge reflexes “.
• Evaluation of energy expenditure by the SenseWear Pro activity monitor (METS)

16 sessions will be held , with the first two reviews pre- training, 14 training sessions totaling eight weeks , twice a week and the last two post-training revaluation . Before the start of each session and after will be three Peak flow measures in the standing position with nose clip .
The groups will be drawn in a sealed envelope in adequate numbers to sample calculation.

REFERENCES

American Association of Cardiovascular & Pulmonary Rehabilitation. Guidelines of pulmonary rehabilitation programmes. 2nd edn. Champaign,IL,USA: Human Kinectics; 1998, ISBN:0-88011-863-6.

American Thoracic Society. Standardization of spirometry; 1994 update. Am J Respir Crit Care Med 1995. 152, 1107–1136.

American Toracic Society, 1999. Recommendations for standardized procedures for the online and offline measurement of exhaled lower respiratory nitric oxide and nasal nitric oxide in adults and children. Am J Respir Crit Care Med.1999;160:2104–2117.

Asher MI, Weiland SK. The international study of asthma and allergy in childhood (ISAAC). ISAAC Steering Committee. *Eur Respir J.*1998;12:315-35.

ATS/ERS Recommendations for Standardized Procedures for the Online and Offline Measurement of Exhaled Lower Respiratory Nitric Oxide and Nasal Nitric Oxide. Am J Respir Crit Care Med. 2005;171:912–30.

Baquet G, Gamelin FX, Mucci P, Thévenet D, Van Praagh E, Berthoin S. Continuous vs. interval aerobic training in 8- to 11-year-old children. J Strength Cond Res. 2010;24:1381-1388.

Baranowski T, Abdelsamad D, Baranowski J, O'Connor TM, Thompson D, Barnett A, Cerin E and Chen TA. Impact of an Active

Video Game on Healthy Children's Physical Activity. Pediatrics 2012 ;129:e636-42.

Barnes PJ, Dweik RA, Gelb AR, Gibson PG, George SC, Grasemann H et al. Exhaled Nitric oxide in pulmonary diseases. A comprehensive review. Chest 2010;138:682-92.

Basaran S, Guler-Uysal F, Ergen N, Seydaoglu G, Bingol-Karakoç G and Altintas DU. Effects of physical exercise on quality of life, exercise capacity and pulmonary function in children with asthma. *J Rehabil Med* 2006; 38:130-135.

Berthoin S, Baquet G, Dupont G, Van Praagh E. Critical Velocity during continuous and intermittent exercises in children. Eur J Appl Physiol.2006;98:132-8.

Biddiss E, Irwin J. Active video game to promote physical activity in children and youth: a systematic review. Arch Pediatr Adolesc Med.2010;164:664-672.

Biddle SJ, Gorely T, Marshall SJ, Murdey I, Cameron N. Physical activity and sedentary behaviours in youth: issues and controversies. J R Soc Promot Health. 2004;124:29-33.

Bonsignore M, RGrutta SL, Cibella F, Scichilone N, Cuttitta G, Interrante A, Marchese M, Veca M, Virzi M, Bonanno A, Profita M, Morici G: Effects of exercise training and montelukast in children with mild asthma.Med Sci Sports Exerc 2008, 40:405-412.

BTS Statement. Pulmonary rehabilitation. *Thorax* 2001; 56:827-834.

Caldeira VS, Starling CCD, Britto RR, Martins JÁ, Sampaio RF e Parreira VF. Precisão e acurácia da cirtometria em adultos saudáveis. *J Bras Pneumol* 2007; 33(5):519-526.

Carson KV, Chandratilleke MG, Picot J, Brinn MP, Esterman AJ, Smith BJ. Physical training for asthma. Cochrane Database Syst Rev. 2013; 30:9.

Carson V, Cliff DP, Janssen X and Okely AD. Longitudinal levels and bouts of sedentary time among adolescents girls. BMC Pediatr.2013;13:173-9.

Cassol VE, Trevisan ME, Moraes EZC, Portela LOC, Barreto SSM. Broncoespasmo induzido pelo exercício em crianças e adolescentes com diagnóstico de asma. *J Bras Pneumol* 2004;30(2)102-108.

Chatkin JM, Djupesland P, Qian W, Haight J, Zamel N. Óxido Nítrico exalado no diagnóstico e acompanhamento das doenças respiratórias. *J Pneumol* 2000; 26(1): 36-43.

Chin A Paw MJM, Jacobs WM, Vaessen EPG, Titze S, Van Mechelen W. The motivation children to play an active video game. J Sci Med Sport. 2008. 11:163-6.

Clark CJ and Cochrane LM. Assessment of work performance in asthma of determination of cardiorespiratory fitness and training capacity.Thorax.1988;43:745-9.

Coelho CC, Aquino ES, Almeida DC, Oliveira GC, Pinto RC, Rezende IMO, Passos C. Análise comparativa e reprodutibilidade do teste de caminhada com carga progressiva (modificado) em crianças normais e em portadoras de fibrose cística. *J Bras Pneumol* 2007;33(2):168-174.

Cohen, J. Statistical power analysis for the behavioral sciences. 2nd ed. Hillsdale (NJ);1988, 567p

Costa D., Fisioterapia Respiratória Básica, São Paulo, Atheneu, 1999.

Counil FP, Varray A, Matecki S, Beurey A, Marchal P, Voisin M and Préfaut C. Training of aerobic and anaerobic fitness in children with asthma. *J Pediatr* 2003; 142:179-184.

Dellaca RL, Ventura ML, Zannin E, Natile M, Pedotti A and Tagliabue P. Measurement of total and compartmental lung volume changes in newborns by optoelectronic pletysmography. *Pediatr Res* 2010: 67:11-16.

Disabella V and Sherman C. Exercise for asthma patients: Little risk, big rewards. Phys Sportsmed.1998;26:75-84.

Dogra S, Kuk JL, Baker J and Jamnik V. Exercise is associated with improved asthma control in adults. Eur Respir J 2011; 37: 318–323.

Donovan CO, Hirsch E, Holohan E, Mc Bride I, Mc Manus R, Hussey J. Energy expended playing Xbox Kinect™and Wii™games: a preliminary study comparing single and multiplayer modes. Physiother 2012;98:224-9.

Drenowatz C, Eisenmann JC. Validation of the SenseWear Armband at high intensity exercise. Eur J Appl Physiol.2011;111:883-7.

Fanelli A, Cabral AL, Neder JA, Martins MA and Carvalho CRF. Exercise training on disease control and quality of life in asthmatic children. *Med Sci Sports Exerc* 2007; 39(9):1474-1480.

Filippelli M, Duranti R, Gigliotti F, Bianchi R, Grazzini M, Stendardi L and Scano G. Overall contribution of chest wall hyperinflation to breathlessness in asthma. *Chest* 2003; 124:2164-2170.

Fujii H, Fukutomi O, Inoue R, Shinoda S, Okammoto H, Teramoto T, Kondo N, Wada H,Saito K, Matsuoka T, Seishima M. Autonomic variability in asthmatic children. *Ann Allergy Asthma Immunol.* 2000 Sep;85(3):233-7.

Gaffin JM, Shotola NL, Martin TR, Phipatanakul W. Clinically Useful Spirometry in preschool aged children: Evaluation of the 2007

American Thoracic Society Guidelines. *J Asthma* 2010 Sep: 47(7):762-767.
Gao Z, Podlog L, Huang C. J Sports Health Science.2013; 2:122-8. Associations among Children's situational motivation, physical activity participation and enjoyment in an active dance video game. J Sports Health Science.2013; 2:122-8.

Gleeson M, Bishop NC, Stensel DJ, Lindley MR, Mastana SS and Nimmo MA. The anti-inflammatory effects of exercise: mechanisms and implications for the prevention and treatment of disease. Nature Review.2011;11:607-615.

Global Strategy for Asthma Management and Prevention, Global Initiative for Asthma (GINA) 2011. Available from: http://www.ginasthma.org/

Goran MI, Kaskoun MC, Carpenter WH, Poehlman ET, Ravussin E, Fontivielle AM. Estimating body composition in young children by using bioelectrical resistance. J Appl Physiol.1993;75:1776-80.

Graf DL, Pratt LV, Hester CN and Short KR. Playing active video games increases energy expenditure in children. Pediatrics 2009;124:534-541.
Hallal PC, Andersen LB, Bull FC, Guthold R, Haskell W, Ekelund U. Global physical activity levels: surveillance progress, pitfalls, and prospects. Lancet Physical Activity Series Working Group. Lancet. 2012;21:380:247-57.

Holloway E, Raw FS. Breathing exercises for asthma. Cochrane Database Syst Rev 2004;(1): CD001277.

Hommerding PX, Donadio MVF, Paim TF and Marostica PJC. The Borg Scale is accurate in children and adolescents older than 9 years with cystic fibrosis. *Respiratory Care* 2010; 55(6):729-736.

International Consensus Report on diagnosis and management of asthma. Bethesda. MD; National Heart Lung and Blood institute. National institutes of Health, 1992; Publication No. 92-3091.

Jardim JR , Mayer AF y Camalier A. Músculos respiratórios y rehabilitación pulmonar en asmáticos. *Arch Bronconeumol* 2002; 38(4):181-188.

Juniper EF, Svensson K, Mörk AC, Ståhl E. Measurement properties and interpretation of three shortened versions of the asthma control questionnaire. Respir Med. 2005;99:553-8.

Kazuma N, Otsuka K, Matsuoka I and Murata M. Heart rate variability during 24 hours in asthmatic children. *Chronobiology International* 1997; 14(6): 597-606.

Kazuma N, Otsuka K, Miyakawa M, Shirase E, Matsuoka I and Murata M. Seasonal variation in heart rate variability in asthmatic children. *Chronobiology International* 2000; 17(4): 503-511.

Ko FWS, Hui DS, Leung T-F, Chu H-Y, Wong GWK, Tung AHM, Ngai JCN, Ng SSS, Lai CKW. Evaluation of the asthma control test: reliable determinant of disease stability and a predictor of future exarcebations. Respirol. 2012;17:370-8.

Kuys SS, Hall K, Peasey M, Wood M, Cobb R, Bell SC. Gaming console exercise and cycle or treadmill exercise provide similar cardiovascular demand in adults with cystic fibrosis: a randomized crossover trial. J Physiother.2011;57: 35–40.

La Scala CSK, Naspitz C, Solé D. Adaptação e validação do Pediatric asthma quality of life questionnaire (PAQLQ-A) em crianças e adolescentes brasileiros com asma. J pediatr (Rio) 2005; 81(1): 54-60.

Lamb KL. Children's Ratings of Effort During Cycle Ergometry: An Examination of the Validity of Two Effort Rating Scales. Pediatr Exerc Scie. 1995; 7: 407-21.

LeBlanc AG, Chaput JP, McFarlane A, Colley RC, Thivel D, Biddle SJ, et al. Active video games and health indicators in children and youth: a systematic review. PLoS One. 2013;8:e65351.

Leite M, Ponte EV, Petroni J, D`Oliveira Júnior A, Pizzichini E, Cruz AA. Evaluation of the Asthma Control Questionnaire validated for use in Brazil. J Bras Pneumol. 2008; 34:756-763.

Li AM,Yin J, Yu CCW,Tsang T, So HK, Wong E, Chan D, Hon EKL and Sung R. The six-minute walk test in healthy children: reliability and validity. *Eur Respir J* 2005;25:1057-1060.

Lopes C, Fonseca J, Silva JPM, Castel-Branco MG. Óxido Nítrico exalado: utilização clínica na asma. Rev Port Imunoalegologia 2005; 13(1): 7-17.

Lopes W A, Rosário N e Leite N. Broncoespasmo induzido pelo exercício em adolescentes asmáticos obesos e não obesos. *Rev Paul Pediatr* 2010;28(1):36-40.

Maddison R, Mhurchu CN, Foley L, Epstein L, Jiang Y, Tsai M, et al. Screen-time weight-loss intervention targeting children at home (SWITCH): a randomized controlled trial study protocol.BMC Public Health. 2011;11:524.

Mc Carthy, Jarret KV and Crawley HF. The development of waist circunference percentiles in British children aged 5,0-16,9y. *European Journal of clinical Nutrition* 2001;55:902-907.

McCormack MC and Enright PL. Making diagnosis of asthma. *Respiratory Care 2008*; 53(5):583-592.

Mellecker RR and Mc Manus AM. Active video games and physical activity recommendations: A comparison of the Gamercize Stepper, XBOX Kinect and XaviX J-Mat. J Sci Med Sport. 2014;17:288-92.

Mendes FA, Almeida FM, Cukier A, Stelmach R, Jacob-Filho W, et al. Effects of aerobic training on airway inflammation in asthmatic patients. Med Sci Sports Exerc. 2011;43: 197-203.

Mendes, F A R; Gonçalves R.C; Nunes M.P.T; Saraiva-Romanholo B. M; Cukier A; Stelmach R; Jacob-Fillho W; Martins MA ; Carvalho C R F. Effects of aerobic training on psychosocial morbidity and symptoms in asthmatic patients: a randomized clinical trial. Chest.2010; 138:331–337.

Miller MR, Hankinson J, Brusasco V, Burgos F, Casaburi R, Coates A, Crapo R, Enright P, et al. Standartisation of spirometry. *Eur Resp J* 2005;26:319-338.

Moreira A, Delgado L, Haahtela T, Fonseca J, Moreira P, Lopes C, Mota J, Santos P, Rytila P, Bastel-Branco MG: Physical training does not increase allergic inflammation in asthmatic children.Eur Respir J 2008, 32:1570-1575.

Ni Mhurchu C, Maddison R, Jiang Y, Jull A, Prapavessis H and Rodgers A. Couch potatoes to jump beans: A pilot study of the effect of active video games on physical activity in children. International Journal of Behavioral Nutrition and physical activity 2008, 5:8 doi: 101186/1479-5868-5-8.

Nóbrega EV, Lima CL, Lima WL, Nobre A, Santos AM, Brito LMO, Costa MRSR. Inspiratory muscle training and respiratory exercises in children with asthma. *J Bras Pneumol* 2008; 34(8):552-558.

Pakhale S, Luks V, Burkett A, Turner L. Effect of physical training on airway inflammation in bronchial asthma: a systematic review. BMC Pulm Med 2013,13:38.

Pedersen BK, Akerstrom TC, Nielsen AR, Fisher CP. Role of myokines in exercise and metabolism. J Appl Physiol. 2007;103:1093-98.

Peyer K, Pivarnik JM, Coe DP. The relationship among HRpeak, RERpeak, and VO2peak during treadmill testing in girls. Res Q Exerc Sport. 2011;82:685-692.

Polgar, P., Promadhat, V., 1971. Pulmonary testing in children. W. B Saunders, Philadelphia, p. 100–153.

Priesnitz CV, Rodrigues GH, Stumpf CS, Viapiana G, Cabral CP, Stein RT, Marostica PJC and Donadio MVF. Reference values for the 6-min walk test in healthy children aged 6-12 years. *Pediatr Pulmonol*. 2009; 44:1174-1179.

Raw FC, Wellington SR, Barnes NC. Inspiratory muscle training for asthma. Cochrane Database Syst Rev 2003; (4): CD003792.

Regamey N, Moeller A. Paediatric exercise testing. *Eur Respir Mon* 2010;47:291-309.

Sarria EE, Rosa RCM, Fischer GB, Hirakata VN, rocha NS, Mattiello R. Versão Brasileira do Paediatric of life questionnaire : Validação de campo. *J Bras pneumol* 2010;36(4):417-424.

Silva CS, Torres LAGMM, Rahal A, Filho JT e Vianna EO. Avaliação de um programa de treinamento físico por quatro meses para crianças asmáticas. *J Bras Pneumol* 2005; 31(4): 279-284.

Smallwood SR, Morris MM, Fallows SJ, Buckley JP. Physiologic responses and energy expenditure of kinect active video game play in schoolchildren. Arch Pediatr Adolesc Med. 2012;166:1005-1009.

Souza VD, Jesus TBA, Souza VF, Dias A, Simões RB, Battagin AM, Costa D, Correa JCF, Oliveira LVF e Sampaio LMM. Efeitos do treinamento físico em crianças asmáticas. *Conscientiae Saúde* 2010; 9(2): 246-252.

Stell IM, Polkey MI, Rees J, Green M, Moxhan J. Inspiratory Muscle strength in acute asthma. *Chest* 2001; 120:757-764.

Torres R. Es possible implementer programas de rehabilitación pulmonary en pediatría? *Neumologia Ped* 2007;2(3):154-158.

Villa F, Castro ABM, Pastorino AC, Santarém JM, Martins MA, Jacob CMA, Carvalho CR. Aerobic capacity and skeletal muscle function in children with asthma. *Arch Dis Child* 2011 doi:101136/adc.2011.212431.

Wanrooj VHM, Willeboordse M, Dompeling E, van de Kant KDG. Exercise training in children with asthma: a systematic review. Br J Sports Med. Published Online 1st: 23 March 2013.

Zijp MHMVC, Ijssestijn H, Takken T, Willemsen SP, Tibboel D, Stam HJ, van den Berg-Emons RJG. Exercise testing of pre-school children using the Bruce treadmill protocol: new reference values. *Eur J Appl Physiol* 2010; 108:393-399.

Zwiren LD. Considerações sobre testes de esforço e sua prescrição durante a infância in Manual de Pesquisa das diretrizes do ACSM (American College Sports medicine) para os testes de esforço e sua prescrição. Guanabara Koogan 4ª Edição, 2003 pag: 522-28.
